# Editorial: Protecting privacy in neuroimaging analysis: balancing data sharing and privacy preservation

**DOI:** 10.3389/fninf.2024.1543121

**Published:** 2025-01-07

**Authors:** Rashid Mehmood, Mariana Lazar, Xiaohui Liang, Juan M. Corchado, Simon See

**Affiliations:** ^1^Faculty of Computer and Information Systems, Islamic University of Madinah, Madinah, Saudi Arabia; ^2^Grossman School of Medicine, New York University, New York, NY, United States; ^3^Department of Computer Science, University of Massachusetts, Boston, MA, United States; ^4^BISITE Research Group, University of Salamanca, Salamanca, Spain; ^5^Air Institute, IoT Digital Innovation Hub, Salamanca, Spain; ^6^Department of Electronics, Information and Communication, Faculty of Engineering, Osaka Institute of Technology, Osaka, Japan; ^7^NVIDIA AI Technology Center, NVIDIA Corporation, Santa Clara, CA, United States

**Keywords:** neuroimaging analysis, privacy-preserving, data sharing, neuroinformatics, artificial intelligence, machine learning, AI explainability, AI governance

Neuroimaging is an indispensable tool in neuroscience and medical research, enabling precise investigations into brain structure and function (Yen et al., 2023; Yan et al., 2022; Shoeibi et al., 2023; Botvinik-Nezer and Wager, 2023; Leite et al., 2024; Wager and Smith, 2003). Techniques such as Magnetic Resonance Imaging (MRI) generate vast amounts of sensitive data, rich in insights yet fraught with privacy challenges (Saponaro et al., 2022; Cali et al., 2023; Li et al., 2020; Zou et al., 2024; Acar et al., 2023). As scientific progress depends on data sharing and collaboration (Martone, 2023), balancing these needs with robust privacy preservation has become a critical concern (Zhang et al., 2020). This Research Topic addresses this challenge by exploring innovative methodologies, frameworks, and technologies that advance the field while safeguarding individual privacy.

The Research Topic aims to promote interdisciplinary research into privacy-preserving solutions for neuroimaging analysis, ensuring compliance with ethical and legal standards (Li et al., 2020). It seeks to balance data utility with privacy protections by fostering methods for anonymization, leveraging AI tools such as federated learning and differential privacy, and aligning technologies with global governance frameworks (Zou et al., 2024; Jeon et al., 2020; Dwork, 2006; Abadi et al., 2016). This Research Topic serves as a roadmap for ethical neuroimaging research and a platform for dialogue among neuroscientists, AI researchers, ethicists, and policymakers.

## Key themes and contributions

This Research Topic features five papers that exemplify the breadth and depth of research at the intersection of privacy, neuroimaging, and artificial intelligence. Each contribution highlights a unique facet of the privacy-preserving landscape, collectively offering a comprehensive exploration of the field's current state and future potential.

The first paper (Cao et al.) tackles the pervasive issue of inflated effect sizes in small-sample neuroimaging studies, a challenge that undermines reproducibility and generalizability. By employing hierarchical Bayesian models, the authors demonstrate how statistical recalibration can improve the reliability of findings while enabling collaborative meta-analyses across studies. This methodological advance sets a foundation for ensuring that shared neuroimaging data is not only secure but also statistically robust.

The second paper (Spahr et al.) explores AI-driven segmentation methods for intracranial hemorrhage detection in CT scans. Leveraging self-supervised and weakly-supervised learning, the study addresses the need for label-efficient solutions that minimize reliance on large, annotated datasets. This work showcases how AI innovations can enhance efficiency and maintain privacy, particularly in resource-constrained environments where data annotation is a bottleneck.

Federated learning takes center stage in the third and fourth papers, both of which highlight its potential for decentralized neuroimaging analysis. The third paper (Mitrovska et al.) introduces a secure federated learning framework for Alzheimer's disease detection, incorporating secure aggregation techniques to protect sensitive data during model training. Similarly, the fourth paper (Thapaliya et al.) presents Sparse Federated Learning for Neuroimaging (NeuroSFL), which optimizes communication efficiency by focusing on sparse sub-networks. Together, these studies underscore the adaptability and scalability of federated learning as a cornerstone of privacy-preserving neuroimaging research.

The final paper (Alsaigh et al.) adopts a broader lens, examining the alignment of AI governance frameworks with neuroinformatics practices. By identifying gaps in existing regulations and proposing strategies for harmonization, the authors provide a roadmap for integrating privacy-preserving technologies within the complex landscape of global governance. This contribution emphasizes the importance of aligning technical advancements with ethical principles, ensuring trust and transparency in neuroimaging research.

## The role of AI and emerging technologies

Artificial intelligence serves as a driving force behind many of the contributions in this Research Topic, offering powerful tools for balancing data sharing and privacy. AI-driven methodologies such as federated learning, differential privacy, and explainable AI not only enable secure data analysis but also enhance transparency and trustworthiness (Yuste, 2023; White et al., 2022; Yang et al., 2022). These technologies address critical challenges, such as mitigating privacy risks in decentralized environments and ensuring that sensitive neuroimaging data remains private without compromising utility.

Federated learning, in particular, emerges as a transformative approach, allowing researchers to train models collaboratively without sharing raw data. The secure aggregation and sparsity-focused innovations presented in this Research Topic demonstrate how federated learning can scale to meet the demands of large, heterogeneous neuroimaging datasets. Complementary technologies such as differential privacy and blockchain also hold promise for further enhancing data security and accountability, though their integration into routine neuroimaging workflows remains a challenge.

## Challenges and open questions

Despite these advancements, significant challenges persist. Balancing data utility with privacy remains a fundamental tension, as techniques that protect privacy often introduce trade-offs in model performance or scalability (Yuste, 2023; Mitrovska et al.). For instance, federated learning frameworks are susceptible to performance degradation in non-IID (non-independent and identically distributed) data settings, a common scenario in neuroimaging. Similarly, the computational demands of privacy-preserving technologies may limit their accessibility to smaller research institutions, exacerbating inequities in the field.

Ethical and societal challenges add another layer of complexity (Aboy et al., 2024; van Kolfschooten and van Oirschot, 2024). Cognitive privacy, informed consent, and equitable access to the benefits of neuroimaging research are ongoing concerns (Bublitz et al., 2024). The rapid evolution of AI often outpaces the development of regulatory frameworks, creating misalignments between technological capabilities and ethical oversight (Ratto Trabucco, 2023; Ienca and Ignatiadis, 2020; Wajnerman Paz, 2022; Jwa and Martinez-Martin, 2024; Yuste et al., 2017; Genser et al., 2024). Addressing these gaps will require sustained dialogue among stakeholders, including neuroscientists, technologists, ethicists, and policymakers (Ligthart et al., 2023; Bublitz et al., 2024).

## Conclusion

This Research Topic highlights the transformative potential of privacy-preserving technologies in neuroimaging, emphasizing the critical balance between advancing data sharing and maintaining individual privacy. By presenting cutting-edge methodologies, practical frameworks, and real-world applications, the contributions collectively offer a comprehensive roadmap for ethical and innovative neuroimaging research. These works demonstrate that privacy and progress can coexist, fostering collaboration and trust across disciplines.

The ubiquity of AI further amplifies the need for dynamic and adaptive regulatory frameworks that evolve alongside technological advancements. As neuroimaging intersects with AI-driven innovation, static and siloed regulations are insufficient to address the complexity of overlapping challenges in data governance and privacy. Instead, flexible approaches that align AI's transformative capabilities with ethical oversight are essential to ensuring responsible progress.

This Research Topic is a testament to the potential of interdisciplinary collaboration in tackling complex challenges at the intersection of technology, ethics, and neuroscience. By fostering dialogue and innovation, it lays the groundwork for a future where neuroimaging research thrives in an environment of trust, transparency, and shared progress. We invite readers to engage with these contributions, advancing the conversation and shaping a more ethical and innovative future for neuroimaging and beyond.

This Research Topic would not have been possible without the dedication of the authors, whose innovative research forms its foundation, the reviewers, whose constructive feedback ensured its rigor, and the neuroimaging and AI communities, whose contributions drive progress in privacy preservation and scientific discovery.
